# Therapeutic role of interleukin-1 receptor antagonist in pancreatic diseases: mendelian randomization study

**DOI:** 10.3389/fimmu.2023.1240754

**Published:** 2023-09-14

**Authors:** Shuai Yuan, Yuyang Miao, Xixian Ruan, Jie Chen, Xue Li, Susanna C. Larsson

**Affiliations:** ^1^ Unit of Cardiovascular and Nutritional Epidemiology, Institute of Environmental Medicine, Karolinska Institutet, Stockholm, Sweden; ^2^ Department of Medicine, Karolinska Institutet, Huddinge, Sweden; ^3^ Department of Geriatrics, Tianjin Medical University General Hospital, Tianjin Geriatrics Institute, Tianjin, China; ^4^ Department of Gastroenterology, Central South University, Changsha, China; ^5^ Department of Big Data in Health Science School of Public Health, Center of Clinical Big Data and Analytics of The Second Affiliated Hospital, Zhejiang University School of Medicine, Hangzhou, China; ^6^ Unit of Medical Epidemiology, Department of Surgical Sciences, Uppsala University, Uppsala, Sweden

**Keywords:** interleukin-1 receptor antagonist, mendelian randomization, pancreatic disease, acute pancreatitis (AP), chronic pancreatitis, pancreatic cancer

## Abstract

**Background:**

The interleukin-1 pathway has been linked to pancreatic diseases. We applied the Mendelian randomization approach to explore whether higher interleukin-1 receptor antagonist (IL-1RA) levels reduce the risk of acute and chronic pancreatitis and pancreatic cancer.

**Methods:**

Genetic variants associated with blood IL-1RA levels at the genome-wide significance level and located 5MB downstream or upstream of the *IL1RN* gene were extracted from a genome-wide meta-analysis of 21,758 participants. After pruning, genetic variants without linkage disequilibrium were used as genetic instrument for IL-1RA. Summary-level data on acute and chronic pancreatitis and pancreatic cancer were obtained from the UK Biobank and FinnGen studies. The associations were meta-analyzed for one outcome from two sources.

**Results:**

Genetically predicted higher levels of IL-1RA were associated with a lower risk of acute and chronic pancreatitis and pancreatic cancer. In the meta-analysis of UK Biobank and FinnGen, the combined odds ratio was 0.87 (95% confidence interval [CI] 0.77-0.97, *P*=0.003) for acute pancreatitis, 0.73 (95% CI 0.65-0.82, *P*=2.93×10^-8^) for chronic pancreatitis, and 0.86 (95% CI 0.77-0.96, *P*=0.009) for pancreatic cancer per one standard deviation increment in genetically predicted levels of IL-1RA.

**Conclusion:**

This study suggests a protective role of IL-1RA in three major pancreatic diseases, which hints the therapeutic potentials of IL-1RA in pancreatic diseases.

## Introduction

Pancreatitis and pancreatic cancer are common disorders of the pancreas and affect over 16 million and four hundred thousand people worldwide, respectively (1, 2). The interleukin 1 (IL-1) pathway has been identified to play a role in the development of pancreatic diseases. Animal studies showed that inhibiting the activity of IL-1β benefited the severity and mortality of acute pancreatitis (3), whereas overexpression of IL-1β induced the development of chronic pancreatitis (4). From the progression of pancreatitis to pancreatic cancer, IL-1β was found to promote tumorigenesis partly via an expansion of immune-suppressive B lymphocytes (5). IL-1β was also found to mediate the association between obesity and pancreatic cancer (6) as well as associate with tumor growth (7) and carcinoma cell migration (8). Even though a few population-based studies linked IL-1 with pancreatic diseases, like an association between IL-1β and a type of pancreatic neoplasms (9), the causality of the associations of IL-1 with the risk of developing pancreatic diseases in humans remains largely uncertain. A clear appraisal of these associations not only deepens the understanding of the pathological basis of pancreatic diseases but also provides evidence support for therapeutic development, such as IL-1 inhibitors (e.g., anakinra).

Mendelian randomization (MR) is an epidemiological design that can reinforce causal inference in an exposure-outcome association by using genetic variants as instrumental variables for the exposure (10). The approach can reduce confounding and reverse causality since genetic variants are randomly assorted at conception and unmodified by onset of the disease (10). Interleukin-1 receptor antagonist (IL-1RA) is an endogenous inhibitor of IL-1 by non-productively binding to IL-1 receptor, which thus prevents both IL-1α or IL-1β from sending signal and thus generating downstream biological effects (11). Genetic variants located in the *IL-1RN* gene are the strongest genetic determinants of blood IL-1RA levels and thus can be used as genetic instruments in MR analysis (12). Here, we conducted an MR investigation to examine the associations of circulating levels of IL-1RA with the risk of three major pancreatic diseases, including acute and chronic pancreatitis and pancreatic cancer.

## Methods

### Study design

**Figure 1:**
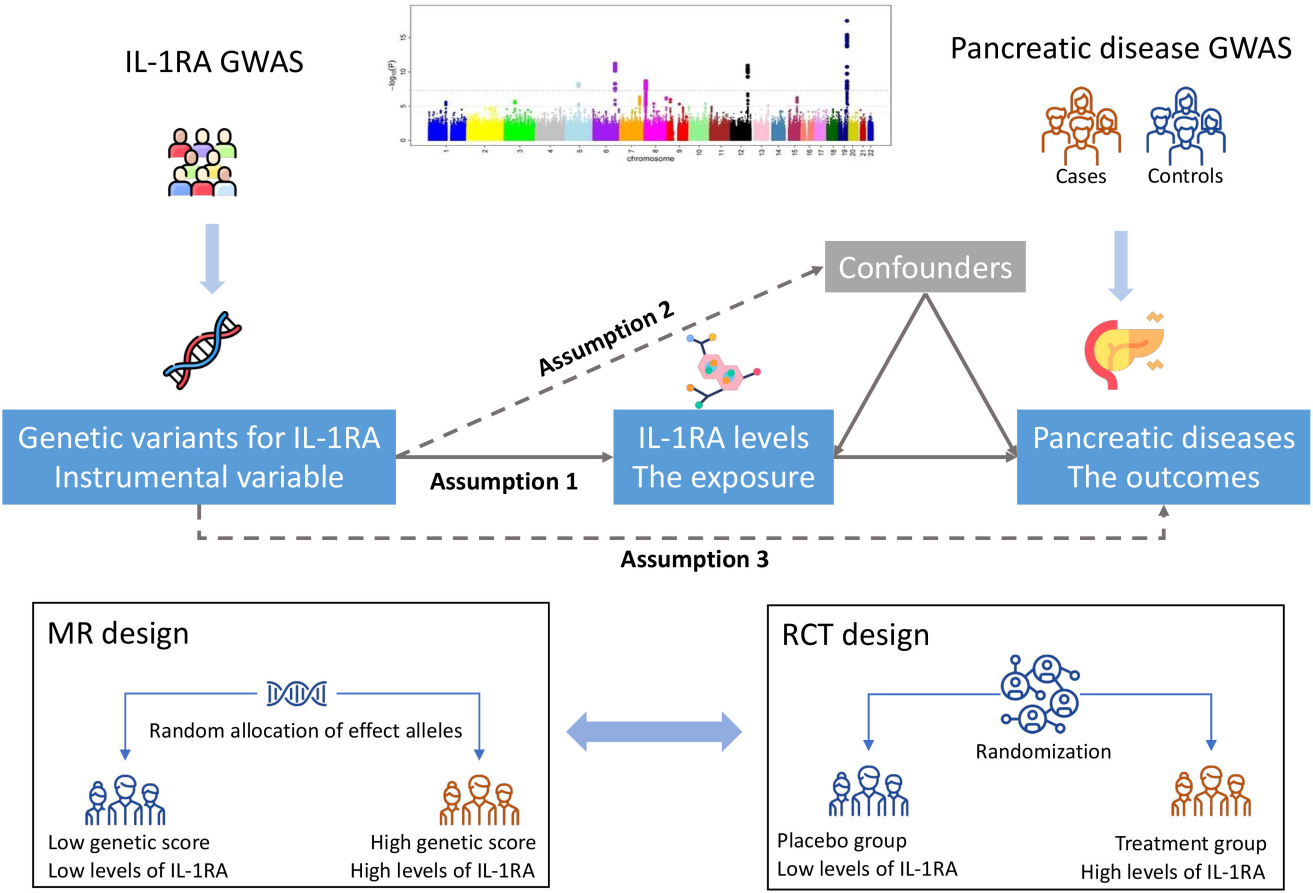
Study design overview. GWAS indicates genome-wide association study; IL-1RA, interleukin-1 receptor antagonist; MR, Mendelian randomization; RCT, randomized controlled trial. There are three key assumptions of Mendelian randomization analysis, which are 1) genetic variants should be robustly associated with the exposure of interest, i.e., blood IL-1RA levels in this study; 2) genetic variants should not be associated with confounders; and 3) genetic variants should not directly associate with pancreatic outcomes or associated with pancreatic outcomes via alternative pathways. The random allocation of effect alleles of MR design resembles the randomization process of RCT, which groups participants into two groups with low and high levels of IL-1RA.

**Figure 1** shows study design overview of the MR design. This MR study has three important assumptions: 1) the employed genetic variants should be strongly associated with blood IL-1RA levels; 2) the used genetic variants should not be associated with any confounder; and 3) the used genetic instruments should influence the risk of pancreatic disease only through the IL-1RA pathway, but not via alternative pathways or directly. This MR study was based on publicly available data from large-scale consortium and cohort studies. Included studies had been approved by corresponding institutional review boards or ethical committees. Participants had signed consent forms. The present MR analyses were approved by the Swedish Ethical Review Authority (2019-02793).

### Genetic instrument selection

Summary-level data on the associations between DNA sequences and levels of IL-1RA were obtained from a genome-wide meta-analysis of up to 21,758 participants from 13 studies (13). The circulating levels of IL-1RA were measured using the Olink CVD I panel with low missing rate caused by below limit of detection or technical problems in included studies. In this genome-wide meta-analysis on blood IL-1RA, the population structure represented by top genetic principal components and study-specific parameters including age, sex, sample storage time, and batch information were adjusted in the genome-wide association analysis (using additive model regressions) in each study where available (13). To select genetic instruments for IL-1RA, we first extracted single nucleotide polymorphisms (SNPs) associated with IL-1RA levels at the genome-wide significance level (*P* < 5×10^-8^) and located in 5MB downstream or upstream of *IL1RN* gene (chromosome 2, GRCh38/hg38 position 113099360-113134014). We then pruned these genetic variants to remove SNPs in high linkage disequilibrium (R*^2^* < 0.1) based on the 1000 Genomics European reference panel. In total, 13 SNPs were used as an instrumental variable for blood IL-1RA levels in MR analysis. The used genetic variants explained approximately 11.6% variance in blood IL-1RA levels. Detailed information on used SNPs is presented in **Table 1**.

**Table 1:** Genetic variants used as instrumental variables for IL-1RA.

rsID	EA	EAF	Beta	SE	*P*	Variance explained
rs1143636	A	0.98	0.242	0.038	1.78E-10	0.37%
rs4591347	T	0.14	-0.101	0.015	1.74E-11	0.42%
rs7584409	A	0.71	-0.086	0.012	9.50E-14	0.50%
rs115652897	T	0.02	-0.251	0.043	3.74E-09	0.32%
rs114189538	A	0.07	-0.167	0.022	6.22E-14	0.52%
rs6743376	A	0.65	-0.182	0.011	3.47E-65	2.60%
rs139076984	T	0.98	0.230	0.040	9.76E-09	0.30%
rs6734238	A	0.61	0.204	0.010	2.46E-85	3.42%
rs74667587	A	0.18	-0.149	0.014	2.69E-26	1.02%
rs72829856	A	0.10	0.213	0.020	2.98E-27	1.07%
rs1491585	A	0.53	-0.075	0.011	1.95E-12	0.45%
rs1191686	T	0.19	-0.072	0.013	4.31E-08	0.28%
rs1867868	T	0.49	0.063	0.011	2.24E-09	0.33%

EA indicates effect allele; EAF, effect allele frequency; IL-1RA, interleukin-1 receptor antagonist; SE, standard error. Beta and SE were scaled to levels of IL-1RA in standard deviation for each additional effect allele.

### Outcome data sources

Summary-level data on the associations of selected IL-1RA-associated SNPs with the risk of acute and chronic pancreatitis and pancreatic cancer were available in the UK Biobank study (14) and the FinnGen study (15). The UK Biobank is an ongoing cohort recruiting over 500,000 participants in the United Kingdom between 2006 and 2010. UK Biobank collected and linked phenotypical, genetic, and health outcome data from questionnaires, physical exams, biological samples, and nationwide health registers. In this study, we used data on acute (1,986 cases) and chronic (514 cases) pancreatitis and pancreatic cancer (589 cases) from genome-wide association studies conducted by Lee lab (16) where these endpoints were defined by codes of the International Classification of Diseases 9th Revision (ICD-9) and ICD-10 (17). The genetic associations with above outcomes were adjusted for sex, birth year, and the first four genetic principal components. The FinnGen study is a nationwide ongoing cohort with genetic and electronic health record data collected. We used the R8 release of FinnGen where acute (5,509 cases) and chronic (3,002 cases) pancreatitis and pancreatic cancer (1,249 cases) were defined by codes of ICD-8, -9 and -10 (17). The genetic associations were adjusted for sex, age, genetic components, and genotyping batch.

### Positive controls

To validate the genetic instruments for IL-1RA, we selected two positive controls. These were C-reactive protein (CRP) and rheumatoid arthritis. CRP is a downstream inflammatory protein of IL-1 pathway. Upregulation of IL-1RA, like by intaking anakinra results in decreased levels of CRP, which has been consistently observed in clinical trials (18, 19). IL-1 inhibitors have been approved to treat rheumatoid arthritis (20). We obtained summary-level data on CRP from a genome-wide association on circulating levels of CRP in 204,402 individuals (21) and data on rheumatoid arthritis from a genome-wide meta-analysis including 19,234 cases and 80,799 controls of European ancestry (22).

### Statistical analysis

The *F*-statistic was estimated to measure the strength of the genetic instrument. An *F*-statistic above 10 indicates a good strength of the genetic instrument. We used the inverse variance weighted method under multiplicative random effects as the primary analysis. In detail, a genetic score was constructed by summing up IL-1RA-increasing alleles weighted by the beta of the allele-IL-1RA associations. The odds ratios of pancreatic diseases were then regressed against this continuous genetic score of IL-1RA, which generated associations scaled to one standard deviation increase in genetically predicted levels of IL-1RA. We combined the association for one outcome from two sources (the UK Biobank and FinnGen) using the fixed-effects meta-analysis method. Although the inverse variance weighted method provides most accurate estimate, it is sensitive to outliers. We therefore conducted three sensitivity analyses, which are the weighted median (23), MR-Egger (24), and Mendelian randomization pleiotropy residual sum and outlier (MR-PRESSO) (25) methods, to test and robustness of the results and to detect and correct for potential horizontal pleiotropy. The weighted median method can provide consistent estimates at the prerequisite that ≥ 50% of the weight comes from valid instrument variants (23). The MR-Egger regression can generate estimates after accounting for horizontal pleiotropy albeit with less precision (24). The MR-PRESSO method can detect SNP outliers and provide results identical to that from the inverse variance weighted analysis after removal of outliers (25). We used Cochran’s Q value to assess the heterogeneity among SNPs’ estimates. MR-Egger intercept test and MR-PRESSO global test were used to detect possible horizontal pleiotropy (*P* < 0.05). We defined the existence of horizontal pleiotropy by significant heterogeneity together with a significant MR-Egger intercept or MR-PRESSO global test. For the association with horizontal pleiotropy, we used the estimate of MR-PRESSO after the removal of outliers and therefore minimized horizontal pleiotropy.

The association with a *P* value <0.017 (0.05/3 outcomes) was deem significant. All analyses were two-sided and performed using the TwoSampleMR and MRPRESSO packages in R software 4.1.2.

## Results

The *F*-statistic ranged from 2607 to 4081 for the studied outcomes, which indicates a good strength of the genetic instrument for IL1-RA. No genetic instruments were strongly associated with the studied pancreatic outcomes. Furthermore, genetically predicted higher levels of IL1-RA were strongly associated with the positive control outcomes, including lower levels of CRP (β = -0.18, 95% confidence interval [CI] -0.22, -0.15, *P* = 2.54×10^-24^) and a decreased risk of rheumatoid arthritis (odds ratio = 0.85, 95% CI 0.78-0.93, *P* = 2.37×10^-4^).

**Figure 2:**
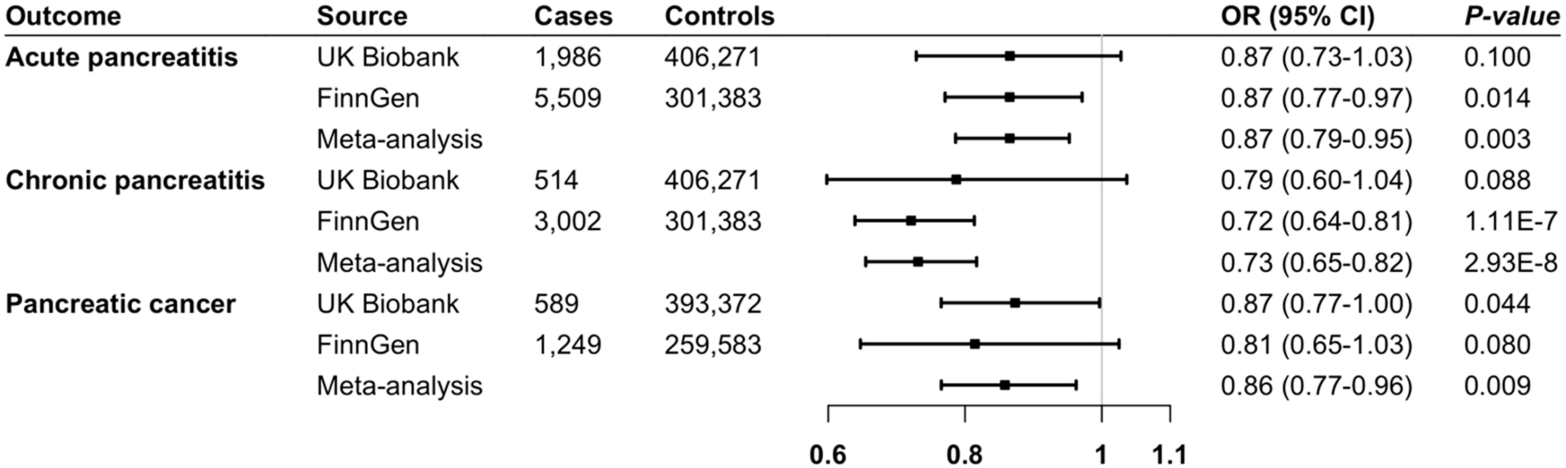
Associations of genetically predicted IL-1RA levels with pancreatic diseases. CI indicates confidence interval; IL-1RA, interleukin-1 receptor antagonist; OR, odds ratio.

**Figure 2** shows the primary results. In the inverse variance weighted analysis (the primary analysis), genetically predicted higher levels of IL-1RA were associated with a non-significant or statistically significant decreased risk of acute and chronic pancreatitis and pancreatic cancer in both UK Biobank and FinnGen. After combing results from two sources, all associations were statistically significant. In the primary analysis, per one standard deviation increment in genetically predicted levels of IL-1RA, the combined odds ratio was 0.87 (95% CI 0.77-0.97, *P* = 0.003) for acute pancreatitis, 0.73 (95% CI 0.65-0.82, *P* = 2.93×10^-8^) for chronic pancreatitis, and 0.86 (95% CI 0.77-0.96, *P* = 0.009) for pancreatic cancer. The associations remained overall consistent in sensitivity analyses (**Table 2**). We observed limited heterogeneity and no outliers across all analyses. MR-Egger intercept and MR-PRESSO global tests detected no indication of potential horizontal pleiotropy (*P* > 0.05). All these together indicated limited horizontal pleiotropy.

**Table 2:** Associations of genetically predicted IL-1RA levels with pancreatic diseases in sensitivity analyses.

Data source	Cases	Controls	Cochran’s Q	*P_Q_*	MR-Egger intercept	*P_intercept_*	Weighted median test			MR-Egger test			MR-PRESSO test
							OR	95% CI	*P*	OR	95% CI	*P*	*P _global test_*
Acute pancreatitis													
UK Biobank	1 986	406 271	13	0.391	0.044	0.155	0.85	0.69-1.06	0.149	0.65	0.44-0.97	0.060	0.470
FinnGen	5 509	301 383	15	0.230	0.012	0.566	0.89	0.77-1.02	0.090	0.80	0.60-1.06	0.153	0.333
Meta-analysis							0.88	0.78-0.99	0.027	0.75	0.59-0.94	0.014	
Chronic pancreatitis													
UK Biobank	514	406 271	8	0.749	0.099	0.106	0.85	0.54-1.32	0.463	0.41	0.19-0.91	0.050	0.661
FinnGen	3 002	301 383	9	0.685	0.018	0.481	0.76	0.63-0.91	0.003	0.64	0.46-0.90	0.025	0.707
Meta-analysis							0.77	0.65-0.91	0.003	0.60	0.44-0.82	0.001	
Pancreatic cancer													
UK Biobank	589	393 372	2	0.999	0.013	0.803	0.95	0.64-1.40	0.779	0.80	0.39-1.66	0.564	0.998
FinnGen	1 249	259 583	14	0.315	-0.024	0.564	0.77	0.57-1.03	0.074	0.95	0.54-1.68	0.869	0.386
Meta-analysis							0.83	0.65-1.04	0.110	0.89	0.57-1.40	0.619	

CI indicates confidence interval; IL-1RA, interleukin-1 receptor antagonist; OR, odds ratio. MR-PRESSO analysis detected no outlier for any associations and thus provided identical results to that of the primary analysis.

## Discussion

This MR study validated the genetic instrument for IL-1RA by examining the associations of genetically proxied IL-1RA levels with CRP levels and rheumatoid arthritis. Using this genetic instrument, we found consistent associations of genetically predicted higher levels of IL-1RA with decreased risk of acute and chronic pancreatitis and pancreatic cancer. These findings indicate the potential therapeutic values of IL-1 inhibitors in treating pancreatic diseases.

Genetically predicted IL-1RA mimics the biological effects of IL-1 inhibitors, which have been used to treat rheumatoid arthritis and cryopyrin diseases with a good tolerance. Afterwards, the utility of IL-1RA inhibitors have been expanded in a broad spectrum of diseases, like to preserve pancreatic islet β-cell function in type 1 diabetes (26). Even though animal studies support a therapeutic effect of IL-1RA on pancreatitis (27–29), few trials have examined the effectiveness of IL-1 inhibitors, like anakinra, in treating pancreatitis or pancreatic cancer. Even though preliminary, this study provided clues that IL-1 inhibitors may be an effective therapeutic target for pancreatic diseases from human genetic perspective. Clinical trials are needed to validate this hypothesis.

There are some potential pathways that may explain the associations of higher levels of IL-1RA and lower risk of pancreatic diseases. IL-1β has been identified as an important cytokine in the development of pancreatitis and pancreatic cancer as well as in the progression from pancreatitis to pancreatic cancer (3–5). High levels of IL-1RA inhibit the signaling of IL-1β by competitively binding to IL-1 receptor, which reduce the risk of developing pancreatic disease, like via B cell-related immune pathways (5) and slower cancer cell growth (7) and migration (8). IL-1 inhibitors have also been found to be protective against inflammation and functional derangement in pancreatic islets (30). In addition, high levels of IL-1 facilitate gallbladder wall inflammation and diminish the absorptive function of the gallbladder epithelium, which may increase the risk of gallstone disease, a risk factor for acute and chronic pancreatitis (31). High levels of IL-1RA may be associated with a good glycemic profile and a low risk of having insulin resistance (32), which has been found to be associated with pancreatic cancer (33).

The advantages of this study include 1) the MR design that minimizes confounding and reverse causation bias; 2) good validation of genetic instruments for IL-1RA indicated by good strength and associations with the positive controls; 3) a comparatively large number of cases by combing data from two large-scale studies; 4) consistent results from two independent populations; and 5) no indication of heterogeneity and horizontal pleiotropy, in particular in this analysis using genetic instruments from the protein encoding gene region. Several limitations deserve discussion. First, this current study was based on data from European population, which confined the generalizability of our findings to other populations. Second, possible nonlinearity and sex-specificity of the associations could not be examined due to lack of individual-level data. Third, this study was based on the general populations. Whether these findings can be generalized to patient groups, such as rheumatoid arthritis patients, need to be verified. In addition, IL-1RA was found to be associated with an increased risk of cardiovascular disease (12, 34). Given an increased risk of cardiovascular disease in rheumatoid arthritis (35), the cardiovascular safety of IL-1 inhibitors among these patients needs to be assessed.

In summary, this study revealed a role of IL-1RA in the development of major pancreatic diseases. Whether IL-1 inhibitors, like anakinra, can be used as a treatment for these disease needs further clinical data.

## Data availability statement

The raw data supporting the conclusions of this article will be made available by the authors, without undue reservation.

## Ethics statement

The studies involving humans were approved by the Swedish Ethical Review Authority (2019-02793). The studies were conducted in accordance with the local legislation and institutional requirements. The participants provided their written informed consent to participate in this study.

## Author contributions

SY and SL conceived and designed the study. SY and XR undertook the statistical analyses. SY wrote the first draft of the manuscript. All authors contributed to the article and approved the submitted version.
